# Dermatofibrosarcoma Protuberans: A Surgeon's Enigma

**DOI:** 10.7759/cureus.44144

**Published:** 2023-08-26

**Authors:** Siva M Gangadhar, Mithilesh Yadav, Nishith S Mandal

**Affiliations:** 1 Surgery, Vardhman Mahavir Medical College and Safdarjung Hospital, New Delhi, IND

**Keywords:** wide excision, local recurrence, chemoradiotherapy (chemo-rt), dermatofibrosarcoma protuberans, immunohistochemistry (ihc)

## Abstract

Dermatofibrosarcoma protuberans (DFSP) is a soft-tissue tumor arising from the dermis. It is a rare tumor, but the important point is that it has a higher incidence of local recurrence than other tumors. Management is primarily by wide local excision (WLE) with tumor negative margin (R0 resection) or Mohs micrographic surgery (MMS). In our case, a 57-year-old male patient presented with an anterior abdominal wall ulcerated mass. The patient had undergone surgery for the excision of the mass, twice before, at a different health-care facility. An incisional biopsy of the mass done at our hospital revealed it to be DFSP. The patient was treated by WLE with flap reconstruction. Post-operative histopathology examination (HPE) report confirmed DFSP with tumor-free margins (R0 resection). So if we fail to diagnose and manage DFSP correctly from the other commonly occurring tumors in the initial stages, there are very high chances of recurrence, and this causes significant morbidity to the patients.

## Introduction

Dermatofibrosarcoma protuberans (DFSP) is a slow-growing, low-grade, metastatic soft-tissue sarcoma, arising from the dermal fibroblast [1]. It is an uncommon tumor, yet it has a significant propensity for local recurrence and invasion of up to 60% [2]. DFSP was first described by Darier and Ferrand in 1924. It was named DFSP by Hoffman in 1925 [3]. It accounts for less than 5% of soft-tissue tumors. It commonly affects the trunk (40%-60%), proximal extremities (20%-30%), and head and neck (10%-15%) [4]. It mostly occurs in middle-aged adults, although congenital forms have been reported in the literature [5]. At the initial presentation, it may be misdiagnosed as lipoma, epidermal cyst, or fibroma. In later stages, differential diagnosis (DD) includes keloid and hypertrophic scar. The standard treatment of DFSP is complete surgical excision with either wide local excision (WLE) with tumor-free margins or Mohs micrographic surgery (MMS). Unresectable or recurrent DFSP are treated with radiation therapy or targeted chemotherapy (CT). We present a case of DFSP on the trunk of a middle-aged male, which was successfully treated by WLE with R0 resection.

## Case presentation

**Figure 1:**
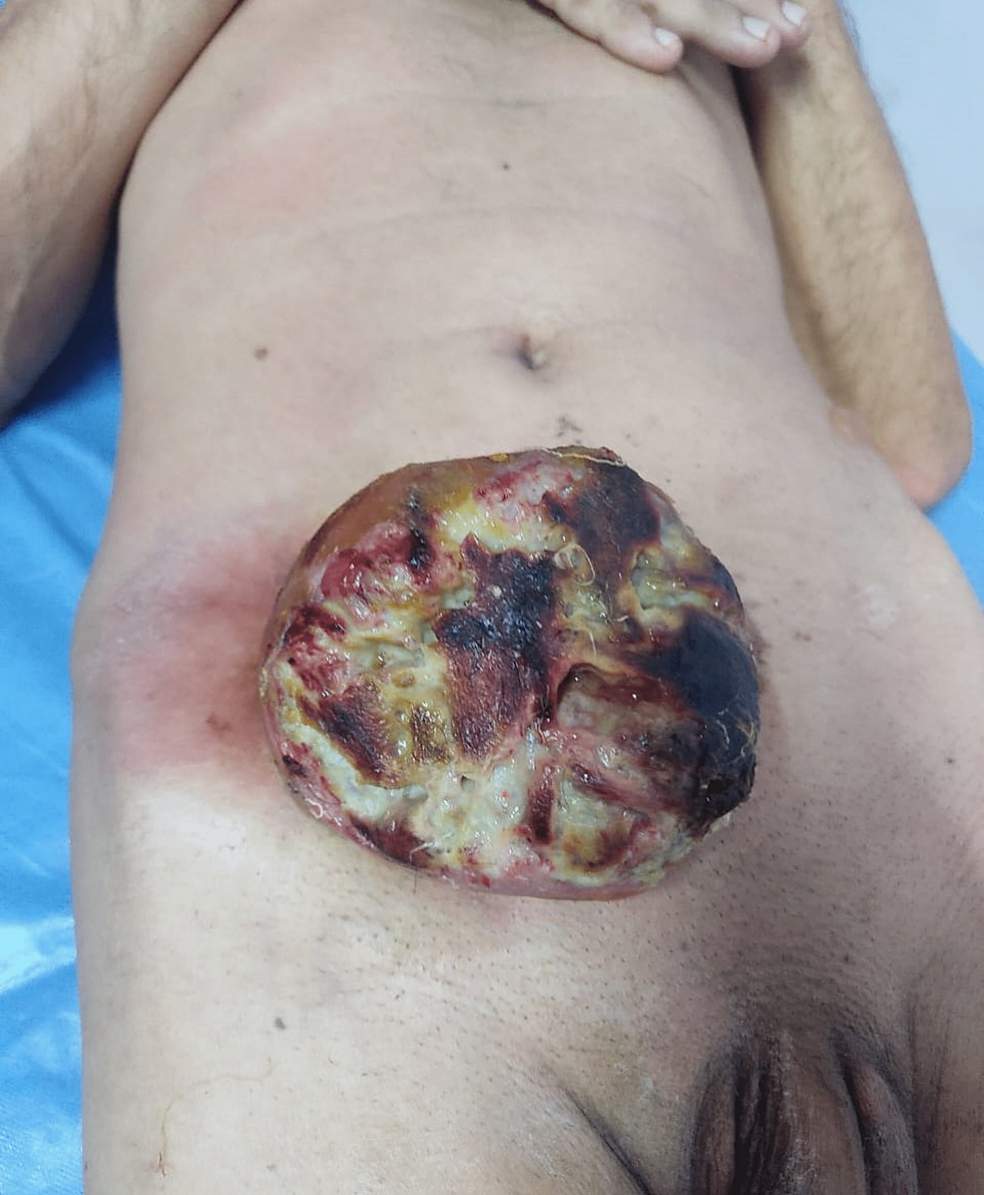
Pre-operative appearance of the tumor

**Figure 2:**
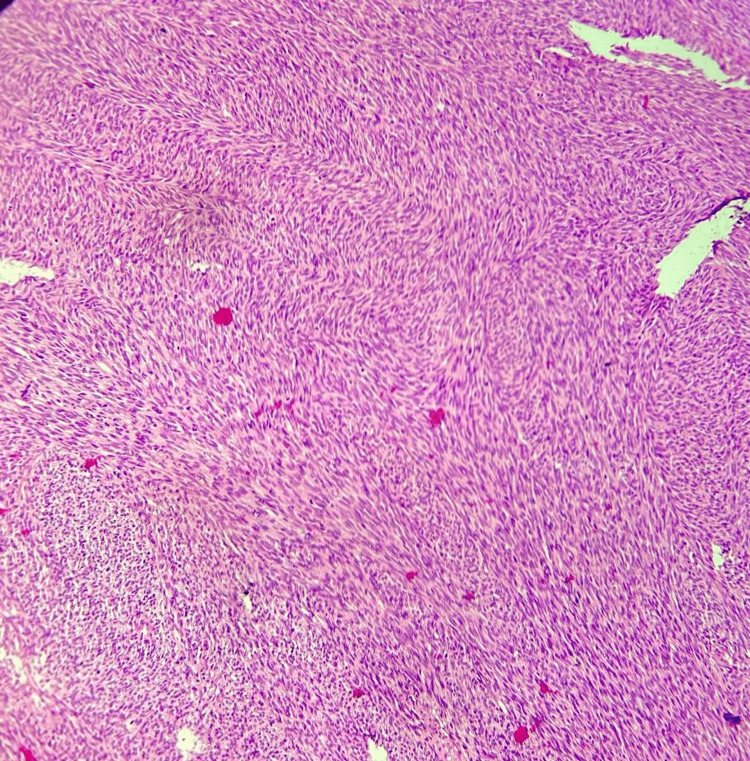
Spindle-shaped tumor cells arranged in fascicle and stratiform pattern as seen under 10× magnification in biopsy

**Figure 3:**
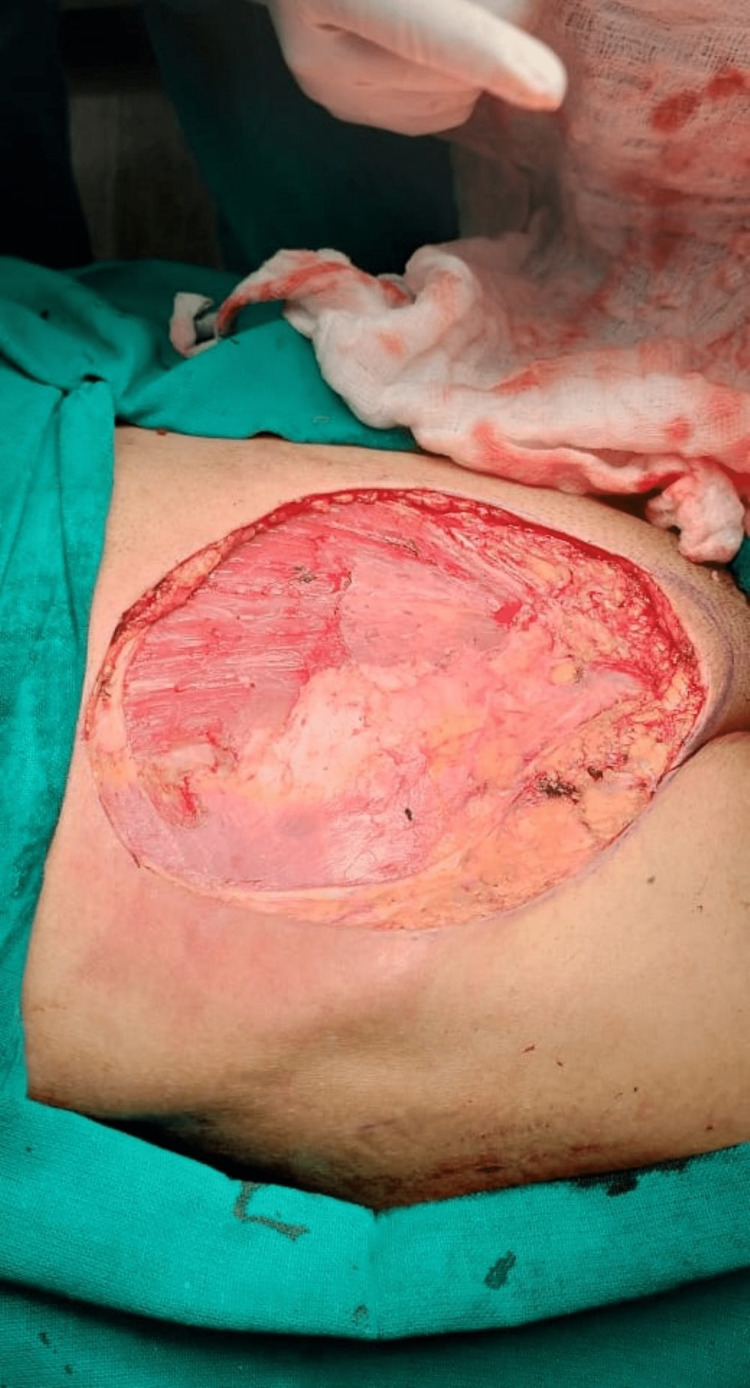
Wide local excision of the tumor, R0 resection

**Figure 4:**
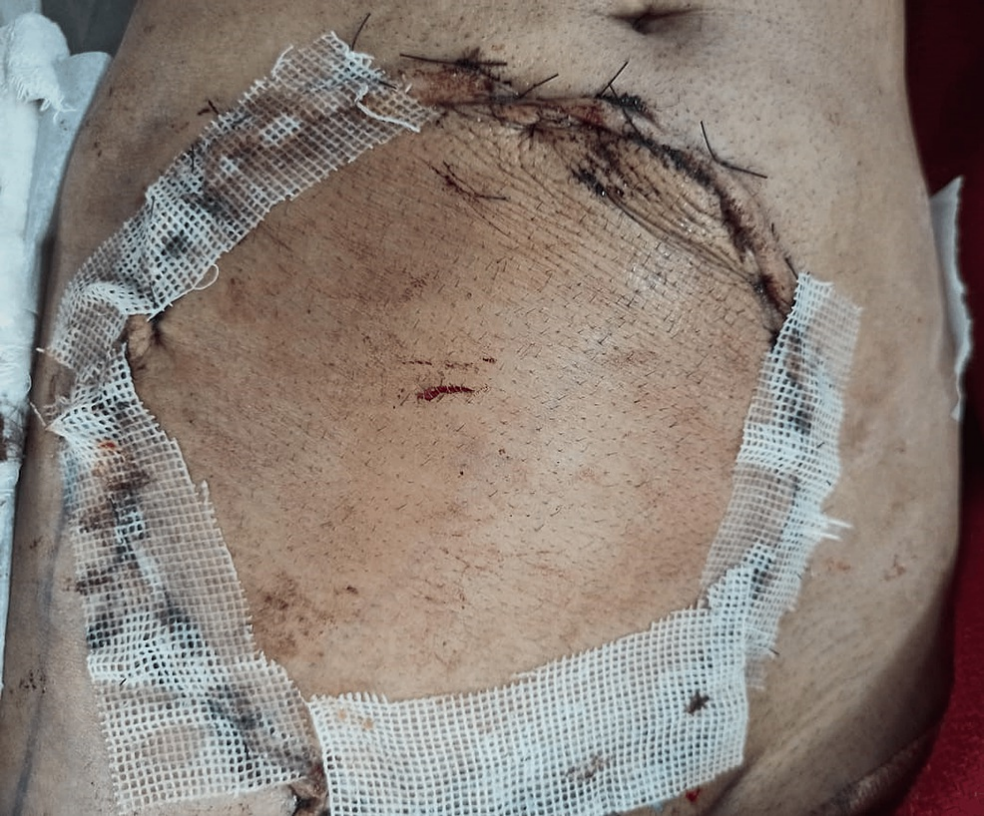
Flap coverage of tumor area

**Figure 5:**
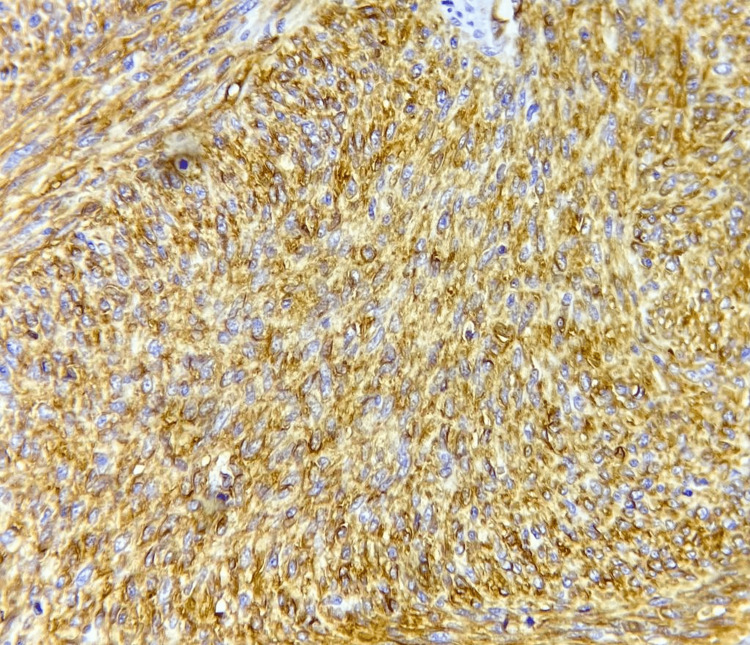
IHC staining showing tumor cells positive for CD34 IHC, immunohistochemistry; CD34, cluster of differentiation 34

A 57-year-old male patient presented to us with a history of anterior abdominal wall mass for four years. The patient had undergone excision of the same mass twice before, in 2020 and 2021, in different hospitals. There was no family history of similar malignancy. The mass was initially small in size, but it gradually progressed to its present size. On physical examination, there was a fixed ulcerated mass of approximately 10×8 cm on the anterior abdominal wall extending into the hypogastrium, umbilical, right iliac, and lumbar regions with overlying slough and necrotic areas and punctuate bleed, without palpable inguinal lymph nodes (Figure 1). Contrast MRI of the abdomen showed a well-defined exophytic mass of 6.6×6.9×6.3 cm abutting the rectus abdominis muscle, without inguinal lymphadenopathy. We did an incisional biopsy, which showed spindle-shaped tumor cells suggestive of DFSP (Figure 2). Tumor staging was done according to the modified European DFSP guidelines, in which the patient was classified as stage II. The patient was planned for wide local excision (WLE) with R0 resection and with anterolateral cutaneous thigh flap and split-thickness skin grafting (Figures 3, 4). Post-operative recovery was uneventful. The final histopathology examination (HPE) report was DFSP. Smears from the tumor showed spindle cells with hyperchromatic nuclei, having eosinophilic cytoplasm. All the resected margins were free of tumors. The tumor cells were positive for cluster of differentiation (CD) 34 on immunohistochemistry (IHC) (Figure 5) and negative for desmin, CD68, and discovered on gastrointestinal stromal tumors 1 (DOG-1). Ki67 proliferation index was 25%. The patient was discussed in the institutional tumor board. No adjuvant CT/radiotherapy (RT) was recommended in view of R0 resection with adequate negative margins without neurovascular/lymphatic involvement and the absence of proven recurrence. The patient was followed up regularly, once every four months for the last two years, and has been disease-free on clinical examination.

## Discussion

DFSP is a low-grade sarcoma arising from the dermal fibroblast. Its incidence is 4.2 per million population. It has an equal male and female preponderance. It occurs between the ages of 20 and 60 years [6]. DFSP occurs mostly in the trunk followed by the proximal extremities and head and neck [7]. DFSP usually presents as a small, firm, non-protuberant, slowly growing, painless, skin-colored dermal plaque or subcutaneous thickening [8]. The early non-protuberant lesions gradually enlarge to form protuberant, indurated, and violaceous nodules later. The external appearance of the tumor belies the true nature of the tumor cells, which frequently invade the underlying soft tissue into the deep fascia, muscle, periosteum, and bones. Distant metastasis is relatively uncommon, mostly to the lungs, brain, other visceral organs, and draining lymph nodes [9].

Pathogenesis

Cytogenetically, it develops due to the chromosomal translocation of t(17;12)(q22;q13) between chromosomes 22 and 17. Translocation leads to the fusion of collage type 1 alpha 1 (COL1A1, at 17q22) and platelet-derived growth factor beta (PDGFB, at 22q13) genes. It results in the dysregulated expression of platelet-derived growth factor beta (PDGFB) protein, tyrosine kinase, which increases tumor cell growth. This understanding has led to the introduction of a new therapeutic lineage of tyrosine kinase inhibitors, imatinib mesylate, for treatment [7].

Differential diagnosis

Differential diagnosis (DD) includes more commonly occurring soft-tissue tumors and fibrolipoma, solitary fibrous tumor, spindle cell lipoma, schwannoma, angiosarcoma, and fibrosarcoma. Misdiagnosis and subsequent inadequate treatment result in high recurrence rates (50%-60%) at three years [10].

Investigations and diagnosis

The definitive diagnosis is by fine-needle aspiration cytology (FNAC), incisional/edge biopsy, and fluorescence in situ hybridization (FISH) or multiplex reverse transcription-polymerase chain reaction (RT-PCR) [11]. On histopathology examination (HPE), it shows a diffuse infiltrate of the spindle cells into the dermis and subcutis with stratiform/cartwheel pattern. Invasion to the adipose tissue shows a honeycomb appearance. DFSP is composed of uniform spindle cells with elongated nuclei and scant cytoplasm surrounded by collagenous stroma. DFSP stains positive for CD34, vimentin, nestin, and apolipoprotein and negative for S100, keratin, desmin, factor XIIIa, and smooth muscle actin (SMA). On the basis of IHC, DFSP has many variants. Histological sub-types include myxoid, Bender tumor, atrophic, sclerosing, and DFSP that have undergone fibrosarcomatous (FS) transformation (FS-DFSP). DFSP without FS component are called classic DFSP, accounting for 80%-90% of all DFSP [12].

Staging

The modified staging system of DFSP is shown in Table 1.

**Table 1:** Staging of DFSP Based on the European consensus interdisciplinary guidelines [13] DFSP: dermatofibrosarcoma protuberans

Stage	Criteria
I	Non-protuberant lesions including atrophic or sclerotic plaque, macula, or small nodules
II	Protuberant lesions
III	Lymph node metastasis
IV	Distant metastasis to other organs

Treatment

Surgery

Management is primarily surgical: conventional excision, WLE (R0 resection), Mohs micrographic surgery (MMS), and partial/total amputation (if the tumor is located on the upper/lower digits). Conventional surgery is done by taking 1 cm of margins around the tumor; however, recurrence is very common. In cases of WLE, a 2-4 cm margin is taken away from the tumor [14]. If DFSP is bigger in size, it may require a reconstructive surgery to cover the defect in the form of local skin flap, skin graft, or a myocutaneous flap [15]. In cases of any doubt about the margin status following excision, we can apply temporary negative pressure dressing and do the flap reconstruction later, after the confirmation of negative margins. An alternative to WLE is MMS [16]. In this procedure, the stepwise horizontal resection of tumor with frozen section is done repeatedly until the report comes out to be tumor-free margins (R0 resection). Both WLE and MMS have different indications in DFSP. WLE is used for DFSP on the trunk and extremities (complete excision feasible in single sitting), while MMS is ideal for DFSP in the head and neck, face, and genitalia (in cosmetically and functionally sensitive regions) [15].

Adjuvant Therapy

Nonsurgical therapy is indicated in locally advanced tumors, recurrent tumors (where it would be difficult to achieve R0 resection), or tumors in which resection is likely to cause unacceptable functional/cosmetic defects. A multidisciplinary specialized soft-tissue sarcoma tumor board approach is recommended [17].

Chemotherapy

Imatinib mesylate is being given for the treatment of inoperable primary/recurrent tumors and metastatic DFSP. It can be given both as neoadjuvant therapy to improve the odds of negative margin and as adjuvant therapy in margin-positive resected patients to prevent recurrence. Response rate varies up to 50%. Moderate dosage of 400-600 mg/day appears to be better tolerated and equally effective as higher dosage of 800 mg/day [18].

Radiotherapy

DFSP is a radio-responsive tumor. Radiotherapy (RT) is an option for primary inoperable tumors, R1/R2 resections, and prior multiple recurrences. Pre-operatively, 50 Gy is given over 25 cycles, and post-operatively, 60 Gy (for microscopic tumors) and 70 Gy (for macroscopic tumors) are given. It is administered by external beam radiotherapy (EBRT), brachytherapy, and intensity-modulated radiation therapy (IMRT). EBRT is given in the form of photons at 1.8-2 Gy/day, five cycles/weekly, and a total dose of 50 Gy is given over 25 cycles over 5-6 weeks. In brachytherapy, multiple radioactive (iridium-192) seeds are given through catheter in the tumor bed. It is given on the fifth post-operative day with a 2 cm margin from the tumor bed. It delivers 42-45 Gy over 4-5 days. Brachytherapy can also be used in cases of recurrent diseases previously treated with EBRT [19].

Prognosis and surveillance

The five- and 10-year recurrence-free survival rates of DFSP (R0 resection) are 86% and 76%, respectively. It is imperative to follow up on these patients in the long run, every 3-6 months during the first three years and annually thereafter, to detect early recurrence. A thorough history and clinical examination of primary site and draining lymph nodes should be done at each visit [20].

## Conclusions

The diagnosis of DFSP is commonly delayed due to the nature of the tumor. We should have a high index of suspicion for DFSP in cases of soft-tissue tumors, and a complete local examination of the swelling along with its draining lymph nodes should be done. Diagnosis can be confirmed by incisional/edge biopsy. HPE report is followed by IHC in suspected cases for differential diagnosis. FISH and RT-PCR are useful tools to confirm a difficult DFSP diagnosis. Metastatic workup is not indicated due to the local invasive nature of the tumor. The complete surgical excision of the tumor (R0 resection) followed by reconstruction (if required) is the treatment of choice. MMS for DFSP on the head and neck and face and WLE for tumors on the trunk and extremities are advocated. Adjuvant therapy, RT, and CT are reserved for recurrent/unresectable tumors. Regular follow-up is recommended to detect early recurrences and for management.
